# hsa_circ_0001776 regulates the progression of acute myeloid leukemia through the miR-1269b/PTEN axis

**DOI:** 10.55730/1300-0152.2769

**Published:** 2025-07-24

**Authors:** Yaoyao WANG, Xiancong YANG, Simin RONG, Xiaoxu LAN, Chenhui TI, Weimiao SUN, Baohui YIN, Youjie LI, Yunxiao SUN

**Affiliations:** 1 Department of Pediatrics, The Second Clinical Medical College, Binzhou Medical University, Yantai, China; 2 Department of Biochemistry and Molecular Biology, Binzhou Medical University, Yantai, China; 3 Laboratory Department, QILU Hospital of ShanDong University DeZhou Hospital, Yantai, China

**Keywords:** hsa_circ_0001776, acute myeloid leukemia, miR-1269b, *PTEN*

## Abstract

**Background/aim:**

Acute myeloid leukemia (AML) is a malignant neoplasm arising from bone marrow hematopoietic stem cells. It is a common subtype of childhood leukemia and remains challenging to cure. Emerging evidence suggests that circular RNAs (circRNAs), a class of noncoding RNAs, play key regulatory roles in tumor biology. Among their various functions, circRNAs often act as ‘sponges’ for microRNAs (miRNAs), modulating gene expression posttranscriptionally. This study investigates the functional role and clinical relevance of hsa_circ_0001776 in AML.

**Materials and methods:**

Three AML cell lines and 22 peripheral blood samples were analyzed. Differential expression analysis of circRNAs in a GSE dataset was performed to identify significantly down- and upregulated candidates, with thresholds set at logFC less than −1 and p < 0.05 for downregulation, and logFC more than 1 and p < 0.05 for upregulation. The back-splice junction of hsa_circ_0001776 was validated using Sanger sequencing. Its circular nature and stability were confirmed via actinomycin D treatment and RNase R digestion. Quantitative real-time PCR (qRT-PCR) was used to measure circRNA levels in clinical AML samples. The functional effects of hsa_circ_0001776 on proliferation and cell cycle progression were evaluated using the CCK-8 assay and flow cytometry. Bioinformatics analyses predicted putative miRNA interactions, which were validated by dual luciferase reporter assays. A p-value less than 0.05 was considered statistically significant.

**Results:**

hsa_circ_0001776 expression was significantly reduced in AML samples. Overexpression of hsa_circ_0001776 inhibited cell proliferation and induced G1 phase arrest in AML cells, whereas knockdown of hsa_circ_0001776 accelerated cell cycle progression and promoted malignant proliferation. hsa_circ_0001776 was shown to interact with miR-1269b, the pair being negatively correlated. *PTEN* was identified as a direct downstream target of miR-1269b, with complementary binding sites confirmed by luciferase assays.

**Conclusion:**

hsa_circ_0001776 suppresses AML progression via the miR-1269b/*PTEN* axis. These findings suggest that hsa_circ_0001776 may serve as a potential diagnostic biomarker and therapeutic target for AML.

## 1. Introduction

Acute myeloid leukemia (AML) is a common and highly heterogeneous malignant neoplasm of the hematopoietic system, characterized by a poor prognosis. Although AML accounts for only 20% of acute leukemia cases in children, it remains a particularly challenging malignancy to treat (Khwaja et al., 2016; Liu et al., 2021). Clinical symptoms are primarily caused by bone marrow hematopoietic stem cell failure, which can result in uncontrolled infections, bleeding, anemia, or infiltration of extramedullary tissues and organs. The pathogenesis of AML is associated with multiple genetic mutations that affect the growth, proliferation, and differentiation of normal blood-forming progenitor cells (Estey and Dohner, 2006). Several novel therapeutic strategies have been developed based on these pathogenic mechanisms, including targeted therapy combined with multidrug regimens, antibody therapy, chimeric antigen receptor T-cell therapy, and drug-based maintenance therapy. Although these approaches have improved patient survival, most individuals have a high risk of relapse (Liu, 2021; Singh et al., 2021; Shao et al., 2023). Recent research has shown that noncoding RNA is a key regulator of AML progression (Wei and Wang, 2015). Therefore, identifying potential therapeutic targets influencing AML and developing effective treatment strategies remain critical research priorities.

Circular RNA (circRNA) is a covalently closed nucleic acid molecule generated by back splicing of precursor messenger RNA (mRNA), and some circRNAs can encode proteins (Zhou et al., 2020; Kristensen et al., 2022; Yang et al., 2022). Unlike linear RNA, circRNA lacks both a 5′-cap and a 3′-poly(A) tail, rendering it resistant to ribonuclease digestion and resulting in greater stability and abundance compared with linear RNA. circRNA is highly enriched and conserved across species (Lei et al., 2020). The functional mechanisms of circRNA include 6 aspects: 1) serving as a sponge for microRNA (miRNA), competitively inhibiting miRNA activity; 2) regulating transcription and translation; 3) sequestering and translocating proteins; 4) acting as a translation template; 5) blocking mRNA translation; and 6) regulating other cells via exosome synthesis (Misir et al., 2022). circRNA is closely associated with the pathogenesis of multiple cancers, with differential expression patterns observed among various cancer types (Ruan et al., 2019; He et al., 2021). For example, circRNA_0025202 acts as a miR-182-5p sponge to regulate breast cancer progression (Sang et al., 2019), whereas circRNA-104718 promotes hepatocellular carcinoma through the microRNA-218-5p/TXNDC5 axis (Yu et al., 2019). circ_0000953 modulates podocyte autophagy in diabetic nephropathy by targeting the mir665-3p/Atg4b axis (Liu et al., 2024), and circ_PPAPDC1A enhances nonsmall cell lung cancer resistance to osimertinib by regulating the miR-30a-3p/IGF1R signaling pathway (Tang et al., 2024). Furthermore, hsa_circ_0001776 is implicated in endometrial cancer and may have clinical relevance for diagnosis and treatment (Ye et al., 2019; Jia et al., 2020).

circRNA regulates tissue development, cell proliferation, innate immunity, and neuronal function. Its expression levels differ among organs. circRNA can interact with proteins, functioning as sponges, decoys, stabilizers, scaffolds, or recruiters (Huang et al., 2020). miRNA is a small, highly conserved noncoding RNA, typically 17–25 nucleotides in length (Lee and Dutta, 2009). Most miRNAs recognize complementary sites within the 3′ untranslated region (3′-UTR) of target mRNAs, thereby suppressing mRNA degradation and translation (Zhang et al., 2019). A single 3′-UTR may contain multiple binding sites for different miRNAs. By regulating downstream target genes, miRNAs participate in various signaling pathways, influencing key biological processes such as cell proliferation, apoptosis, differentiation, autophagy, and immune response (Tang et al., 2015). The function of miRNAs is context dependent, determined by the cellular environment and target genes, and they may act as tumor suppressors or oncogenes (Iorio and Croce, 2009). For example, miR-93-5p inhibits ovarian cancer progression by targeting SLC7A11 and increasing ferroptosis (Li et al., 2024), whereas miR-99a-3p promotes gastric cancer development by suppressing TRIM21 expression (He et al., 2024).

Although circRNA has diverse functions as a noncoding RNA, research on circRNA in leukemia remains limited. hsa_circ_0001776 is downregulated in endometrial cancer tissues and cells, and its functional mechanism has been investigated. By analyzing the leukemia-related dataset GSE116617, we identified hsa_circ_0001776, which has not previously been studied in AML. To explore the function of hsa_circ_0001776 in AML, we collected clinical blood samples and confirmed that hsa_circ_0001776 is downregulated in AML. These findings suggest that hsa_circ_0001776 may be a potential biomarker for AML diagnosis and treatment.

## 2. Materials and methods

### 2.1. Patients and samples

Blood samples were collected from 11 patients with AML and 11 healthy controls at Yantai Affiliated Hospital of Binzhou Medical University. All participants received a comprehensive informed consent document, ensuring that patient rights were fully respected and protected. The study received formal approval from the Ethics Committee of Binzhou Medical University (code: 2022-603, 20 December 2022) and was conducted in strict accordance with medical ethical standards.

### 2.2. Bioinformatics analysis

The public dataset GSE116617 was obtained from the Gene Expression Omnibus (GEO) database^[1]^. According to the dataset description, GSE116617 contains 4 healthy control and 8 AML samples. Grouping information was obtained using the pData function in R software (version 4.2.1), and subsets were extracted. After setting the reference level, differential expression was analyzed with the limma package. Differential circRNA expression was visualized using a heatmap generated with R software and a volcano plot was created using GraphPad Prism (version 8.0). The sequence of hsa_circ_0001776 was retrieved from circBase^[2]^. Downstream miRNAs were predicted using circBank^[3]^. And the binding sites between hsa_circ_0001776 and miRNAs were analyzed using both circBank and the bioinformatics platform^[4]^. miRNA–mRNA regulatory sites were identified using TargetScan^[5]^ and miRDB^[6]^. The regulatory network was visualized with Cytoscape (version 3.8.2), and the STRING database^[7]^ was used to identify core hub genes. Functional enrichment analysis of these hub genes was performed using the Kyoto encyclopedia of genes and genomes (KEGG) pathway database.

### 2.3. Cell culture and treatments

AML cell lines THP-1, HL-60, and U937, as well as the human embryonic kidney cell line HEK-293T, were obtained from the Cell Bank of the Chinese Academy of Sciences (Shanghai, China). THP-1, HL-60, and U937 cells were cultured in RPMI 1640 medium (Pricella, Wuhan, China), and HEK-293T cells were maintained in Dulbecco’s modified Eagle’s medium (Pricella, Wuhan, China). All media were supplemented with 10% fetal bovine serum (Gibco, Thermo Fisher Scientific, Waltham, MA, USA) and 1% penicillin–streptomycin (Beyotime Biotechnology, Shanghai, China). Cells were incubated in a humidified atmosphere at 37 °C with 5% CO_2_.

### 2.4. Sanger sequencing

RNA was extracted from cells and converted to complementary DNA (cDNA) using a reverse transcription kit (Vazyme Biological Co., Ltd., Nanjing, China). Polymerase chain reaction (PCR) was performed under the following conditions: initial denaturation at 95 °C for 1 min, followed by 40 cycles of 95 °C for 20 s, 60 °C for 30 s, and 72 °C for 30 s. Amplified products were subjected to agarose gel electrophoresis, and the resulting bands were sent to Qingdao Qingke Biological Company for Sanger sequencing.

### 2.5. RNA extraction, reverse transcription, and quantitative real-time PCR

Peripheral blood samples from AML patients and healthy controls were processed to separate blood cells and serum. For RNA extraction, 3 times the volume of red blood cell lysis buffer (Biosharp, Hefei City, China) was added to blood cells, mixed gently, and incubated for 15 min to lyse erythrocytes. After centrifugation at 12,000 rpm for 6 min, the supernatant was removed. Total RNA was extracted from the remaining cells and AML cell lines using TRIzol reagent (Thermo Fisher Scientific). cDNA synthesis was performed with the PrimeScript RT reagent kit (Takara Bio, Kusatsu, Shiga, Japan) using 1 μg of total RNA as template. Quantitative real-time PCR (qRT-PCR) was conducted with SYBR PCR Master Mix (Vazyme Biotech). The cycling conditions were: initial denaturation at 95 °C for 3 min, followed by 40 cycles of 95 °C for 20 s, 60 °C for 20 s, and 72 °C for 20 s. Relative expression and fold change were calculated using the 2^−ΔΔCt^ method, with *GAPDH* as the internal control. Primer sequences are provided in Table 1.

### 2.6. RNase R assay

Total RNA (<5 μg) extracted from AML cells was treated with 1–3 U/μg of RNase R (20 U/μL) at 30 °C for 30 min in a final volume of 20 μL, with RNase-free water as the mock control. An internal reference without RNase R treatment was used as a normalization control. After digestion, RNase R was inactivated at 70 °C for 10 min. The expression of circRNA and linear RNA was evaluated by qRT-PCR.

### 2.7. Laboratory instruments

The principal laboratory instruments used in this study are listed in Table 2.

### 2.8. Actinomycin D

AML cell lines were treated with 2 μg/mL actinomycin D (GENESEED, Guangzhou, China) for 4, 8, 12, and 24 h to inhibit RNA transcription. Untreated cells served as negative controls. A total of 1 × 10^5^ cells per well were seeded in a 12-well plate. Total RNA was extracted at each time point and subjected to qRT-PCR to assess the stability of hsa_circ_0001776 and *ESYT2*.

### 2.9. Vector construction, lentiviral packaging, and cell infection

Lentiviral vectors were constructed by Research Cloud Biology (Jinan, China). For lentiviral packaging, HEK-293T cells at 70–80% confluency were starved for 1 h. Plasmids were transfected at a ratio of 7.5:5.5:2 μg (target plasmid, PAX2, and MD2G (v = m/c)). After 5 min at room temperature, 20 μL ExFect Transfection Reagent (Vazyme Biotech Co., Ltd.) was added and mixed. After 24 h, the culture medium was replaced, and the virus-containing supernatant was collected, filtered through a 0.45 μm filter, and centrifuged at 8000 rpm for 30 min at 4 °C. The pellet was resuspended. THP-1, HL-60, and U937 cells were seeded in 6-well plates and cultured at 37 °C with 5% CO_2_. When the cell density reached 65–80%, cells were infected with lentivirus, and the medium was changed 8 h postinfection. After 24 h, changes in green fluorescent protein expression were evaluated using fluorescence microscopy and flow cytometry, and images were acquired.

### 2.10. Agarose gel electrophoresis

Divergent and convergent primers (Qingdao Vazyme Biotech) were synthesized to amplify cDNA and genomic DNA (gDNA), respectively, from AML cell lines. gDNA was extracted using a commercial kit (TIANGEN, Beijing, China). qRT-PCR was performed with the following cycling parameters: 98 °C for 10 s, 55 °C for 30 s, and 72 °C for 1 min, for a total of 30 cycles. To analyze PCR products from gDNA and cDNA, 1% agarose gel electrophoresis was conducted using 1× tris–acetate–EDTA buffer. DNA fragments were separated at room temperature at 110 V for 35 min, and bands were visualized using a gel imaging system.

### 2.11. CCK-8 assay

To assess cell proliferation, THP-1 and U937 cells infected with lentivirus were seeded into 96-well plates (7000 cells per well) and cultured at 37 °C with 5% CO_2_. At 0, 24, 48, and 72 h after transfection, 10 μL of CCK-8 solution (Beyotime Biotechnology) was added to each well and incubated for 2 h. Absorbance at 450 nm was measured using a microplate reader.

### 2.12. Cell cycle analysis

After 72 h of lentiviral infection, THP-1 and U937 cells cultured in 6-well plates were collected and centrifuged at 1200 rpm for 4 min. Cells were fixed in 1 mL of 75% ethanol at 4 °C and stored overnight at 4 °C or −20 °C. Before staining, cells were washed with phosphate-buffered saline (PBS), and 100 μL of RNase A solution was added to the pellet. The suspension was incubated at 37 °C for 1 h, followed by the addition of 400 μL of propidium iodide staining solution. Samples were incubated in the dark at 4 °C for 1 h. Cell cycle distribution was analyzed by flow cytometry, and data were processed using FlowJo software (version 10.8.1).

### 2.13. Antibodies

Rabbit antihuman GAPDH antibody (1:6000, catalog number: AP0063, Bioworld Technology, Inc., Nanjing, China), *PTEN* rabbit polyclonal antibody (1:1000, catalog number: 9559S, Cell Signaling Technology, Leiden, Netherlands), and goat antirabbit immunoglobulin G (IgG)–horseradish peroxidase (HRP) secondary antibody (1:6000, catalog number: L27A9, Bioworld Technology, Inc.) were used in this study.

### 2.14. Dual luciferase assay

Wild-type (WT) and mutant (Mut) DNA fragments containing the predicted miR-1269b binding sites in hsa_circ_0001776 and *PTEN* were amplified and cloned into the pcDNA3.1-luci vector. The recombinant plasmids (WT-pcDNA3.1-luci-hsa_circ_0001776, Mut-pcDNA3.1-luci-hsa_circ_0001776, WT-pcDNA3.1-luci-*PTEN*, and Mut-pcDNA3.1-luci-*PTEN*) were constructed. For the assay, HEK-293T cells were seeded into 6-well plates (1 × 10^5^ cells/well, 70–80% confluency) for 24 h before transfection. Cells were cotransfected with the appropriate luciferase plasmid and either the plv-miR-1269b vector or plv-NC control. After 48 h, firefly luciferase (FL) and renilla luciferase (RL) activities were measured using a dual luciferase detection kit (Vazyme Biotech Co., Ltd.) following the manufacturer’s instructions. The FL to RL activity ratio was used to evaluate the interaction between miR-1269b and hsa_circ_0001776 or *PTEN*.

### 2.15. Western blot analysis

AML cells were seeded in 6-well plates and transfected with either plv-miR-1269b or plv-NC. After 48 h, cells were washed twice with PBS, then lysed with 150 μL radioimmunoprecipitation assay buffer containing protease inhibitors (Beyotime Biotechnology). Lysates were incubated on ice for 30 min, then sonicated for 10 min. Supernatants were collected after centrifugation. Protein samples were separated by 10% sodium dodecyl sulfate–polyacrylamide gel electrophoresis and transferred to polyvinylidene fluoride membranes. Membranes were blocked in 5% milk at 37 °C for 2–4 h, followed by overnight incubation at 4 °C with primary antibodies (rabbit antihuman GAPDH, 1:6000, Bioworld Technology; *PTEN* (138G6) rabbit mAb, 1:1000, Cell Signaling Technology). After washing with 1× TBST 3 times (20 min each), membranes were incubated with goat antirabbit IgG (H + L) HRP at 4 °C for 2 h. Chemiluminescent reagents were used to visualize protein bands.

### 2.16. Statistical analysis

Data were analyzed using GraphPad Prism (version 8.0) and SPSS (IBM SPSS Statistics 26). Receiver operating characteristic (ROC) curve analysis was performed with GraphPad Prism, and comparisons between 2 groups were conducted using independent sample t-tests. Statistical significance was set at p < 0.05. Significance levels are indicated as follows: ns, not significant; *p < 0.05; **p < 0.01; ***p < 0.001; ****p < 0.0001.

## 3. Results

### 3.1. hsa_circ_0001776 is downregulated in AML

**Figure 1:**
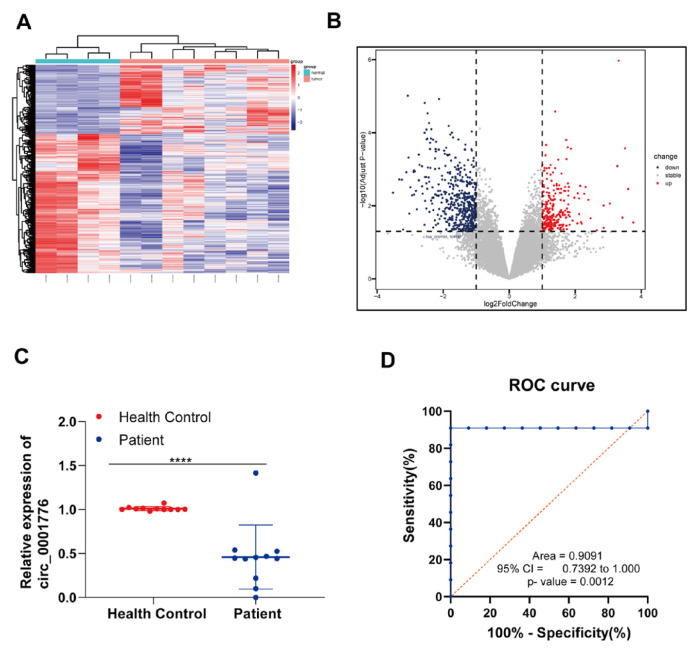
circ_0001776 is downregulated in AML. A) For the GSE116617 dataset, differentially expressed circRNAs are presented in the form of heatmaps. B) Volcano plot of differentially expressed circRNAs in GSE116617 (blue represents downregulation, red represents upregulation, gray represents no significant difference. C) The expression level of circ_0001776 was examined by qRT-PCR in healthy control (n = 11) and AML patients (n=11). D) ROC curve analysis of circ_0001776. ****p < 0.0001.

Previous studies have reported that hsa_circ_0001776 is downregulated in endometrial cancer. To assess its expression in leukemia, we analyzed the GSE116617 dataset from the GEO database and found that hsa_circ_0001776 (also known as hsa_circRNA_104547) was significantly downregulated in leukemia samples. Heatmaps and volcano plots illustrate these findings (Figures 1A and 1B).

To validate these observations, we performed qRT-PCR analysis on blood samples from 11 AML patients and 11 healthy controls. The results confirmed that hsa_circ_0001776 expression was significantly lower in AML patients compared with controls (Figure 1C). Additionally, ROC curve analysis showed an area under the curve (AUC) of 0.9091 (p = 0.0012) for hsa_circ_0001776, indicating substantial diagnostic value (Figure 1D). Collectively, these data suggest that hsa_circ_0001776 may serve as a prognostic biomarker and therapeutic target for AML.

### 3.2. Identification and characterization of hsa_circ_0001776 in AML

**Figure 2:**
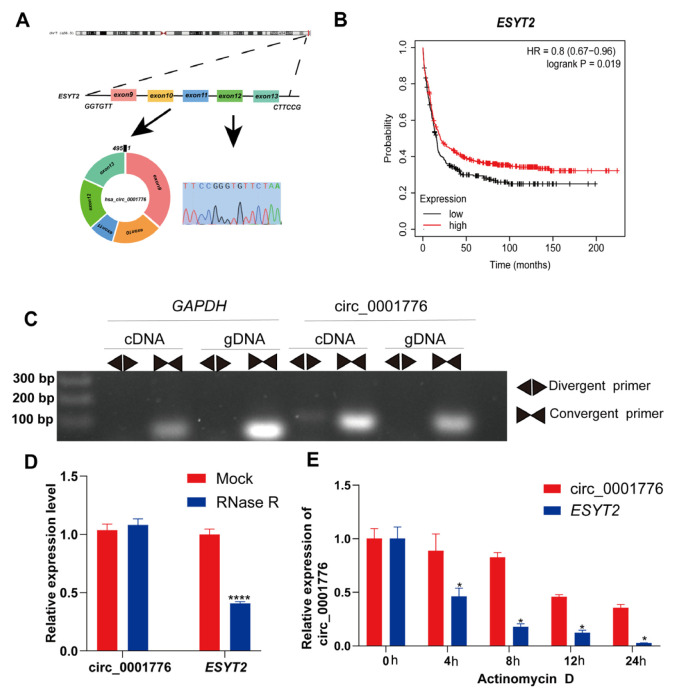
Origin and characteristics of circ_0001776. A) A diagram showing the origin of circ_0001776 and the identification of the connection point of circ_0001776 by Sanger sequencing. B) Kaplan–Meier survival curve analysis of the maternal gene *ESYT2* of circ_00017766. C) The existence of circ_0001776 was verified by agarose gel electrophoresis and gel electrophoresis with convergent or divergent primers. D) The expression levels of circ_0001776 and *ESYT2* mRNA in AML cells treated with or without RNase R were detected by real-time fluorescence quantitative PCR. E) Real-time fluorescence quantitative PCR showed that the abundance of circ_0001776 and *ESYT2* mRNA in AML cells after treatment with actinomycin D at a specified time point. *p < 0.05, ****p < 0.0001.

The existence of hsa_circ_0001776 was confirmed by Sanger sequencing. hsa_circ_0001776 is derived from the *ESYT2* gene and consists of spliced exons 9–13, located at chr7:158,552,176–158,557,544 (Figure 2A). The mature sequence is 495 nucleotides in length. Kaplan–Meier survival analysis indicated that patients with AML and high *ESYT2* gene expression had higher overall survival rates (Figure 2B). To further validate the existence of hsa_circ_0001776, divergent primers were designed to amplify circular transcripts, whereas convergent primers were used to detect linear transcripts from cDNA and gDNA. cDNA and gDNA were isolated from U937 cells and analyzed by agarose gel electrophoresis. Divergent primers amplified circular products from cDNA but not from gDNA, whereas convergent primers amplified linear products from both cDNA and gDNA (Figure 2C). We also investigated the stability of hsa_circ_0001776 in AML cells. Total RNA was pretreated with RNase R, and hsa_circ_0001776 showed greater stability than linear *ESYT2*, further confirming its circular structure (Figure 2D). U937 cells were treated with actinomycin D, a transcription inhibitor, and total RNA was collected at specified time points. Analysis of hsa_circ_0001776 and *ESYT2* expression showed high stability of the circRNA subtype (Figure 2E). These results show that hsa_circ_0001776 is a stably expressed circRNA in human AML cells.

### 3.3. hsa_circ_0001776 affects cell proliferation and cell cycle in AML

**Figure 3:**
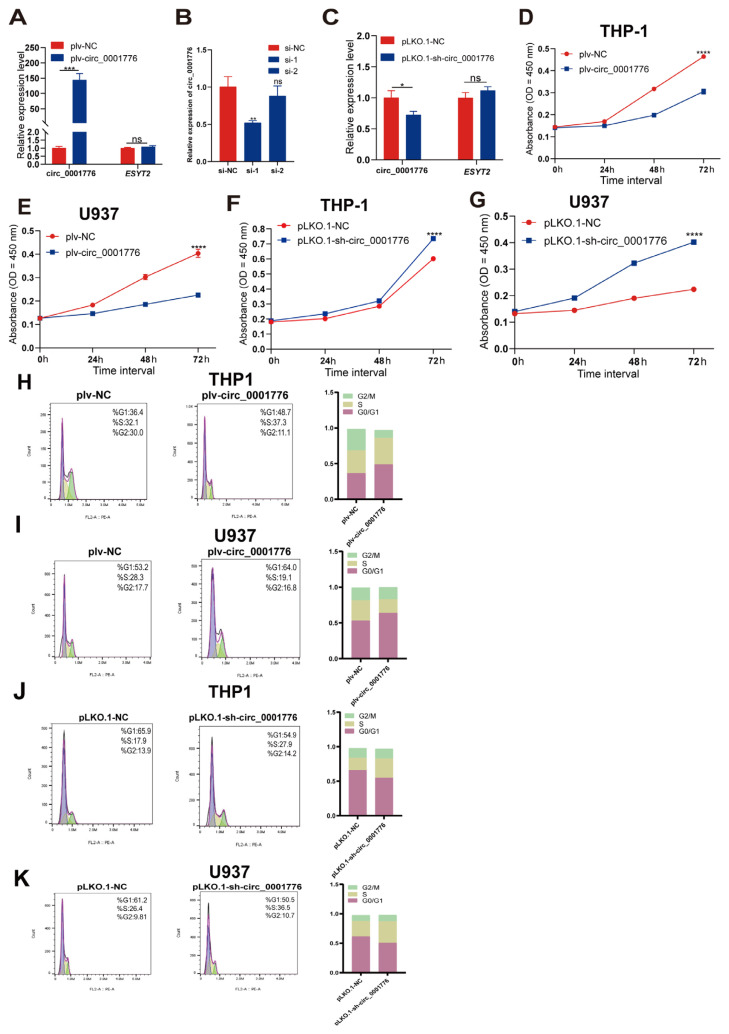
circ_0001776 inhibits the proliferation of AML cells and Circ_0001776 affects the cell cycle progression of AML. A) The expression levels of circ_0001776 and *ESYT2* mRNA in AML cells after overexpression of circ_0001776 were detected by real-time fluorescence quantitative PCR. B) Real-time fluorescence quantitative PCR was used to detect the expression level of siRNA or si-NC after stable transfection into AML cells. (C) The expression levels of circ_0001776 and *ESYT2* mRNA in AML cells after stable transfection of pLKO.1-sh-circ_0001776 or pLKO.1-NC were detected by real-time fluorescence quantitative PCR. D) and E) The effects of plv-NC and plv-circ_0001776 on the proliferation of AML cell lines were determined by CCK-8. F) and G) The effects of pLKO.1-NC and pLKO.1-sh-circ_0001776 on the proliferation of AML cell lines were determined by CCK-8. H) and I) The cell cycle progression of AML cells after overexpression of circ_0001776 was analyzed by flow cytometry. The number of cells represents the percentage of cells. J) and K) The cell cycle progression of AML cells after knockdown of circ_0001776 was analyzed by flow cytometry. The number of cells represents the percentage of cells. *p < 0.05, **p < 0.01, ***p < 0.001, ****p < 0.0001.

To investigate the function of hsa_circ_0001776 in AML, we constructed a vector (plv-circ_0001776) for effective overexpression in AML cells. A small interfering RNA (siRNA) fragment was designed for hsa_circ_0001776 knockdown, and 2 siRNAs targeting hsa_circ_0001776 were transfected into HEK-293T cells. qRT-PCR analysis of transfection efficiency showed that si-hsa_circ_0001776-1 had the highest efficiency. This fragment was selected to construct a vector (PLKO.1-sh-circ_0001776) for lentiviral packaging. The overexpression and knockdown efficiency of hsa_circ_0001776 was validated by qRT-PCR. Compared with the control vector, siRNA significantly reduced hsa_circ_0001776 expression, whereas the overexpression plasmid increased its expression in AML cells (Figures 3A–3C). CCK-8 assays showed that overexpression of hsa_circ_0001776 significantly inhibited proliferation of THP-1 and U937 cells (Figures 3D and 3E), whereas knockdown of hsa_circ_0001776 significantly increased proliferation of these cells (Figures 3F and 3G).

We further assessed cell cycle changes by flow cytometry. Overexpression of hsa_circ_0001776 increased the percentage of cells in the G0/G1 phase and significantly decreased the percentage in the G2/S phase. Conversely, knockdown of hsa_circ_0001776 reduced the G0/G1 population and significantly increased the G2/S phase percentage. Compared with the control, overexpression of hsa_circ_0001776 slowed the cell cycle, whereas knockdown accelerated it. These results indicate that hsa_circ_0001776 exerts an anticancer effect in AML cells (Figures 3H–3K).

### 3.4. hsa_circ_0001776 acts as a competitive endogenous RNA for miR-1269b

**Figure 4:**
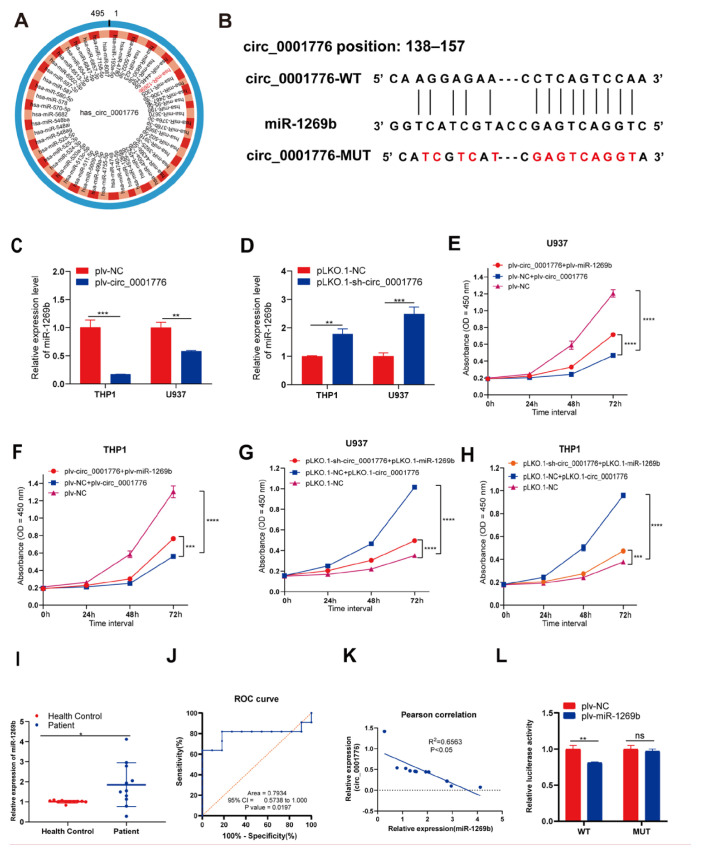
circ_0001776 serves as sponge for miR-1269b. A) circ_0001776 absorbs downstream miRNA map. B) The binding site map of circ_0001776 and miR-1269b. C) The expression level of miR-1269b in AML cells after overexpression of circ_0001776 was detected by real-time fluorescence quantitative PCR. D) Real-time fluorescence quantitative PCR was used to detect the expression level of miR-1269b in AML cells after knocking down circ_0001776. E) and F) Overexpression of miR-1269b rescued the inhibitory effect of circ_0001776 overexpression on cell proliferation by CCK-8 assay. G) and H) Knockdown of miR-1269b rescued the effect of pLKO.1-sh-circ_0001776 on the proliferation of AML cells by CCK-8 assay. I) The expression level of miR-1269b in 11 pairs of AML patients and healthy controls was detected by real-time fluorescence quantitative PCR. J) Receiver operating characteristic curve of miR-1269b. K) Correlation analysis between miR-1269b and circ_0001776. L) The interaction between hsa_circ_0001776 and miR-1269b was evaluated by dual luciferase reporter gene assay. *p < 0.05, ***p < 0.001, ****p < 0.0001.

To further elucidate the mechanism of hsa_circ_0001776 in AML, we utilized circBank to predict candidate miRNA that may interact with hsa_circ_0001776. The analysis identified 52 miRNA as potential targets of hsa_circ_0001776. Previous studies have reported that miR-1269b is upregulated in cancer (Kong et al., 2016; Lv et al., 2022). Therefore, miR-1269b was selected for subsequent investigation (Figure 4A). In addition, we predicted the specific binding site between hsa_circ_0001776 and miR-1269b (Figure 4B).

To examine whether hsa_circ_0001776 modulates tumor progression via miR-1269b, we performed rescue experiments in AML cell lines. qRT-PCR results showed that, compared with the control group, miR-1269b expression was significantly reduced in hsa_circ_0001776-overexpressing U937 and THP-1 cells. In contrast, miR-1269b expression was significantly increased in hsa_circ_0001776-underexpressing U937 and THP-1 cells (Figures 4C and 4D).

To further clarify the role of hsa_circ_0001776 in AML cells, we constructed vectors to achieve miR-1269b overexpression (plv-miR-1269b) and knockdown (PLKO.1-miR-1269b). Subsequently, plv-circ_0001776, plv-miR-1269b, or both were transfected into U937 and THP-1 cells. The CCK-8 assay showed that overexpression of miR-1269b reversed the inhibitory effect of hsa_circ_0001776 overexpression on cell proliferation (Figures 4E and 4F). Similarly, transfection of PLKO.1-sh-circ_0001776, PLKO.1-miR-1269b, or both into U937 and THP-1 cells indicated that knockdown of miR-1269b could reverse the promoting effect of hsa_circ_0001776 knockdown on cell proliferation (Figures 4G and 4H).

We also conducted qRT-PCR analysis on clinical blood samples from patients with AML and healthy controls. The results showed that the expression level of miR-1269b was higher in patients with AML than in normal individuals (Figure 4I). To evaluate the diagnostic potential of miR-1269b, we constructed ROC curves that yielded an AUC of 0.7934 (p = 0.0197) (Figure 4J). Furthermore, miR-1269b expression was negatively correlated with hsa_circ_0001776 levels in AML (Figure 4K).

The interaction between hsa_circ_0001776 and miR-1269b was validated by a dual luciferase assay. The luciferase activity of cells cotransfected with hsa_circ_0001776-WT and plv-miR-1269b was significantly lower than that of the control group. However, the luciferase activity of cells cotransfected with hsa_circ_0001776-MUT and plv-miR-1269b did not change significantly, and no statistical significance was observed (Figure 4L).

### 3.5. Expression of miR-1269b in AML and its downstream target PTEN

**Figure 5:**
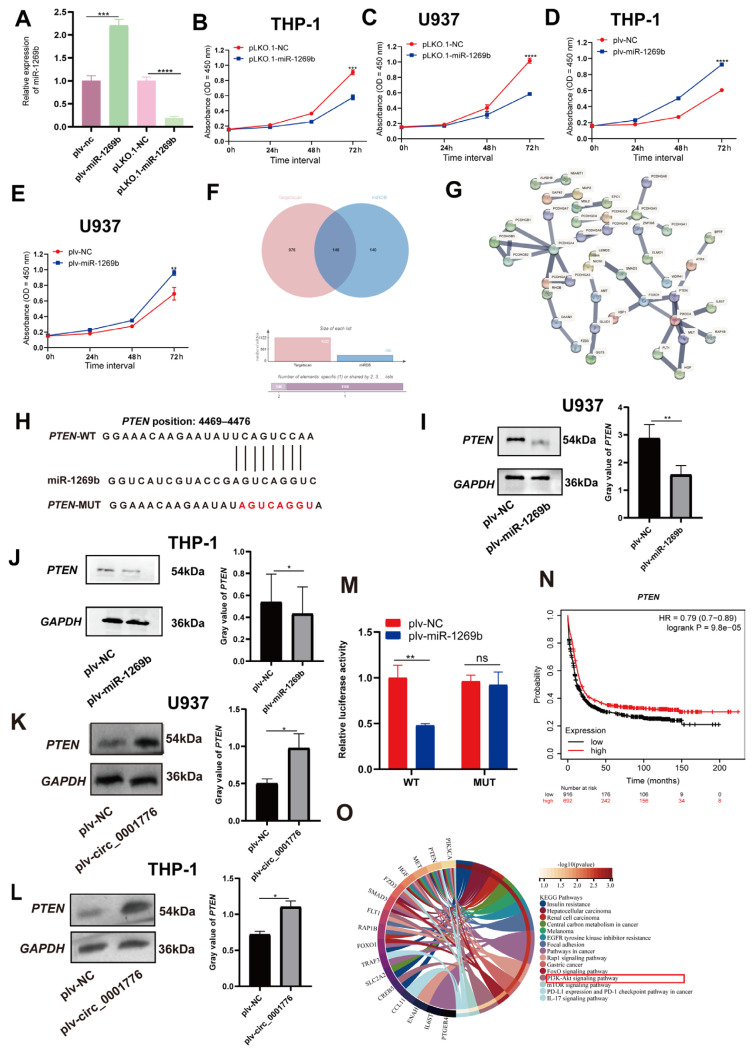
miR-1269b affects AML cell proliferation and *PTEN* is a direct target of miR-1269b. (A) The efficiency of miR-1269b overexpression and knockdown was detected by real-time fluorescence quantitative detection in AML cells. B) and C) The effect of miR-1269b knockdown on the proliferation of AML cells was detected by CCK-8 assay. D) and E) The effect of miR-1269b overexpression on the proliferation of AML cells was detected by real CCK-8. F) The overlapping target genes of miR-1269b were predicted by TargetScan and miRDB. G) PPI protein interaction network was constructed by STRING database. H) Binding sites between miR-1269b and *PTEN*. I) and J) Western blot was used to detect the relative expression of *PTEN* in AML cells overexpressing miR-1269b. K) and L) Western blot was used to detect the relative expression of *PTEN* in AML cells overexpressing hsa_circ_0001776. M) The interaction between miR-1269b and *PTEN* was evaluated by dual luciferase reporter gene assay. N) Kaplan–Meier survival curve of *PTEN*. O) KEGG functional enrichment analysis of miR-1269b. *p < 0.05, **p < 0.01, ***p < 0.001, ****p < 0.0001.

To validate the efficiency of miR-1269b overexpression and knockdown, AML cells were infected with plv-miR-1269b and PLKO.1-miR-1269b, respectively. qRT-PCR analysis showed that miR-1269b expression significantly increased in plv-miR-1269b-infected cells and significantly decreased in cells treated with PLKO.1-miR-1269b compared with controls (Figure 5A).

AML cell lines U937 and THP-1 were transfected with plasmids to overexpress or knock down miR-1269b, and cell proliferation was assessed using a CCK-8 assay. Consistent with the observed upregulation of miR-1269b in AML, knockdown of miR-1269b reduced proliferation relative to controls (Figures 5B and 5C), whereas overexpression of miR-1269b enhanced proliferation (Figures 5D and 5E).

To investigate the mechanism of miR-1269b action in AML, potential target genes were predicted using TargetScan and miRDB. Intersection analysis identified 146 candidate targets (Figure 5F). Construction of a protein–protein interaction network with STRING further characterized these candidates (Figure 5G). TargetScan predicted a binding site between miR-1269b and *PTEN* (Figure 5H). Western blot analysis showed that overexpression of miR-1269b downregulated *PTEN* gene levels in both U937 (Figure 5I) and THP-1 cells (Figure 5J). In contrast, overexpression of hsa_circ_0001776 in U937 (Figure 5K) and THP-1 (Figure 5L) cells upregulated *PTEN* protein expression. Dual luciferase reporter assays showed that cotransfection with *PTEN*-WT and plv-miR-1269b resulted in significantly decreased luciferase activity compared with controls, whereas *PTEN*-MUT cotransfection had no significant effect (Figure 5M).

Kaplan–Meier survival analysis showed that higher *PTEN* expression was associated with improved overall survival in AML patients (Figure 5N). KEGG functional enrichment analysis was conducted to further explore the biological functions and signaling pathways associated with *PTEN* (Figure 5O). Collectively, these results show that miR-1269b directly targets *PTEN*, and hsa_circ_0001776 regulates *PTEN* expression in AML cells by functioning as a sponge for miR-1269b.

### 3.6. hsa_circ_0001776 regulates PTEN expression via mir-1269b sponging

**Figure 6:**
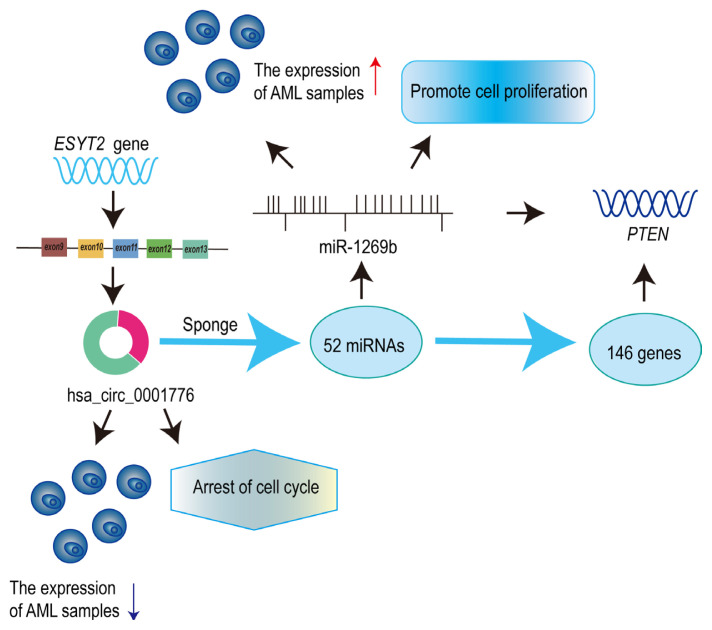
hsa_circ_0001776 regulates *PTEN* expression by acting as a sponge for miR-1269b.

The mechanism of action is illustrated in Figure 6. hsa_circ_0001776, derived from exons of its parental gene *ESYT2*, suppresses proliferation and cell cycle progression in AML cells by sponging miR-1269b and modulates AML progression by regulating the downstream target gene *PTEN*.

## 4. Discussion

With the advancement of medical research in recent decades, significant progress has been achieved in the diagnosis and treatment of pediatric AML. However, relapse and mortality rates remain high due to the rare and deadly nature of AML in children (Zwaan et al., 2015; Rasche et al., 2018; Fleischmann et al., 2021). Therefore, identifying effective treatments and reliable therapeutic targets to improve prognosis remains crucial (Vago and Gojo, 2020; Bewersdorf and Abdel-Wahab, 2022). Only by fully understanding the molecular pathogenesis of AML can improved therapeutic targets be identified. Research on circRNA has highlighted its important role in human diseases. The characteristics and functions of circRNA make it a promising diagnostic biomarker and therapeutic target, with a growing range of potential applications (Vausort et al., 2016; Chen et al., 2021; Hansen et al., 2022).

circRNA is a novel type of RNA formed by the back splicing of exons, which connects the ends of exons. It has a relatively long half-life and high conservation, and it is involved in various biological processes (Zhang et al., 2023). circRNA not only regulates gene expression but also contributes to the pathogenesis of several diseases (Geng et al., 2018). An increasing number of circRNAs are reported to be associated with tumorigenesis. Some circRNAs are differentially expressed in tumor and normal tissues, which may provide new clinical insights for tumor diagnosis and treatment (Lin et al., 2022). For example, hsa_circ_0015278 may serve as a novel prognostic marker for *FLT3*-ITD mutation and poor prognosis in AML (Jiang et al., 2022). CircSLC25A13 promotes AML progression through the miR-616-3p/ADCY2 axis, providing new perspectives for AML therapy (Wei et al., 2023). CircAFF2 regulates AML cell function by binding PML mRNA (Yao et al., 2025). CircTADA2A stabilizes p53 expression by interacting with TRIM28, thereby inhibiting the progression of *FLT3*-ITD acute myeloid leukemia (Li et al., 2025). CircBMI1 acts as a tumor suppressor gene in AML by regulating miR-338-5p/ID4, and may affect the pathogenesis of AML through exosome secretion (Su et al., 2024). Circ_0001187 regulates AML progression through the miR-499a-5p/RNF113A/METTL3 axis and acts as a key tumor suppressor in AML (Yang et al., 2023). CircROBO1 promotes the development and liver metastasis of breast cancer through the circROBO1/KLF5/FUS feedback loop. This loop inhibits the selective autophagy of afadin by suppressing *BECN1* transcription (Wang et al., 2022). CircROBO1 also contributes to enzalutamide resistance and glycolysis in prostate cancer through the circROBO1–miR-556-5p–PGK1 axis (Zhou et al., 2023). CircCOCH affects liver cancer progression by regulating miR-450a and activating the PI3K/mTOR pathway (Jiang et al., 2024). hsa_circ_0101050 promotes colon cancer development by targeting the miR-140-3p/MELK axis (Cheng et al., 2024). Studies show that exosome-derived circ_0006896 limits the antitumor immune response by interacting with HDAC1, thereby promoting AML progression (Can et al., 2025). Circ_0035381 promotes AML progression by regulating the miR-186-5p/CDCA3 pathway (Xu et al., 2025). In this study, bioinformatics data were obtained from the GEO database and analyzed with multiple bioinformatics platforms to minimize errors and provide reliable results. Experimental data were analyzed using GraphPad Prism, and clinical data were analyzed using SPSS. We predicted downstream miRNA and targets with various platforms to construct a competing endogenous RNA (ceRNA) regulatory network.

In the experiment, we verified the existence of hsa_circ_0001776 and further explored the mechanism by which hsa_circ_0001776 functions, confirming that hsa_circ_0001776 acts as a sponge for miRNA. In addition, we constructed different vectors for hsa_circ_0001776 and transfected them into AML cells to verify their effect on AML cell proliferation. Subsequently, we used bioinformatics to construct a ceRNA regulatory network to show that miR-1269b is a key downstream molecule of hsa_circ_0001776 in our model. Some reports have confirmed that miR-1269b interacts with certain target genes and its abnormal expression plays a crucial role in the metastasis and recurrence of certain cancers, participating in the regulation of disease-related physiological processes in the human body. For example, miR-1269b regulates the expression of SVEP1, affecting the proliferation and metastasis of hepatocellular carcinoma (Chen et al., 2020). miR-1269b influences the cell proliferation, migration, and invasion of gastric cancer by regulating the expression of METTL3 (Kang et al., 2021). The tumor suppressor function of PTEN is evident in childhood and adult cancers (Chen et al., 2018). Studies have found that PTEN is lowly expressed in AML (Esmaeili et al., 2021). Moreover, circ_0002232 transcribed from *PTEN* regulates the progression of AML through the miR-92a-3p/PTEN network (Su et al., 2020). In addition, the inhibition of PRL2 can up-regulate the expression of PTEN protein and improve the progression of AML (Carlock et al., 2023). HOTAIR inhibits PTEN expression by up-regulating the DNMT3b-dependent mechanism and induces doxorubicin resistance in AML (Zhou et al., 2021). We studied the negative correlation between the expression of miR-1269b and *PTEN* in AML cells. Our results indicate that hsa_circ_0001776 can act as a sponge for miR-1269b, affecting the expression of downstream *PTEN* mRNA. hsa_circ_0001776 can be further studied as an potential therapeutic target for pediatric AML.

There are several limitations in this study. The exploratory analysis was conducted in only 11 AML patients, and the limited sample size makes the ROC curve analysis less robust. Although a potential association of hsa_circ_0001776 and miR-1269b with clinical outcomes was observed, this finding should be considered preliminary and used to generate hypotheses. The stability of the model requires validation in larger cohorts. The role of *PTEN* and its signaling pathway in AML has not yet been investigated. Future research will assess the effect of *PTEN* on AML cell proliferation, apoptosis, and differentiation by knocking down or overexpressing *PTEN*. This study lacks in vivo validation, which can be addressed in future research by establishing animal models.

The hsa_circ_0001776/miR-1269b/*PTEN* axis affects AML progression by regulating cell proliferation and the cell cycle. Circular RNA can adsorb miR-1269b and relieve its inhibition of the tumor suppressor *PTEN*, thereby slowing AML progression. For diagnosis, hsa_circ_0001776 and miR-1269b may serve as stable biomarkers. For prognosis, low hsa_circ_0001776 or high miR-1269b indicates *PTEN* deletion and is associated with poor prognosis.

## Figures and Tables

**Table 1:** Primer sequences used in the experiment.

Primers	Sequences (5′-3′)
hsa_circ_0001776-FP	TCAAACCTCGACAAGGTGCT
hsa_circ_0001776-RP	CCTTAGAACACCCGGAAGGT
ESYT2-FP	CAAACTATCTGGTGCTTCCCAA
ESYT2-RP	GGAAACCGCAACTGAGCTATT
GAPDH-F(Divergent)	GAAGACTGTGGATGGCCCCT
GAPDH-R(Divergent)	CAAATGAGCCCCAGCCTTCT
GAPDH-F(Convergent)	GCTGAACGGGAAGCTCACTG
GAPDH-R(Convergent)	GTGCTCAGTGTAGCCCAGGA
miR-1269b-FP	CTGGACTGAGCCATGCTACTGG
miR-1269b-RP	CGAACATGTACAGTCCATGGATAG
U6-F	CTCGCTTCGGCAGCACA
U6-R	AACGCTTCACGAATTTGCGT
si-1	CAAGGAACCTTCCGGGTGT
PTEN-FP	TGGATTCGACTTAGACTTGACCT
PTEN-RP	GCGGTGTCATAATGTCTCTCAG

**Table 2:** The main instruments used in the experimental process.

Instrument name	Source
Thermostatic cell incubator	Thermo USA
Real time fluorescent quantitative PCR	Thermo Company, USA
Flow cytometry	BD FACSCalibur, Inc.
Western blot electrophoresis apparatus	Bio-rad, Inc.

## Data Availability

The data generated in the present study may be requested from the corresponding author and first author. The data are not publicly available due to reasons pertaining to patient privacy.

## Footnotes

1 NCBI (2017). GEO database [online]. Website https://www.ncbi.nlm.nih.gov/geo/ [accessed 11 October 2025].

2 circBase (2014). circBase [online]. Website http://www.circbase.org/ [accessed 11 October 2025].

3 circBank (n.d.). circBank [online]. Website http://www.circbank.cn/ [accessed 11 October 2025].

4 bioinformatics platform [online]. Website http://www.bioinformatics.com.cn/ [accessed 11 October 2025].

5 TargetScan (n.d.). TargetScan [online]. Website http://www.targetscan.org [accessed 11 October 2025].

6 miRDB (2020). miRDB [online]. Website http://www.mirdb.org [accessed 11 October 2025].

7 STRING (n.d.). STRING [online]. Website https://cn.string-db.org/ [accessed 11 October 2025].
